# A Pyranoxanthone as a Potent Antimitotic and Sensitizer of Cancer Cells to Low Doses of Paclitaxel

**DOI:** 10.3390/molecules25245845

**Published:** 2020-12-10

**Authors:** Fábio França, Patrícia M. A. Silva, José X. Soares, Ana C. Henriques, Daniela R. P. Loureiro, Carlos M. G. Azevedo, Carlos M. M. Afonso, Hassan Bousbaa

**Affiliations:** 1 CESPU, Institute of Research and Advanced Training in Health Sciences and Technologies (IINFACTS), University Institute of Health Sciences (IUCS), Rua Central de Gandra, 1317, 4585-322 Gandra, Portugal; fabiofuff@gmail.com (F.F.); patricia.silva@cespu.pt (P.M.A.S.); A24955@alunos.cespu.pt (A.C.H.); 2 Department of Biomedical Sciences and Medicine, University of Algarve, 8005-139 Faro, Portugal; 3 LAQV-REQUIMTE, Department of Chemical Sciences, Laboratory of Applied Chemistry, Faculty of Pharmacy, University of Porto, Rua de Jorge Viterbo Ferreira, 228, 4050-313 Porto, Portugal; jfxsoares@ff.up.pt; 4 Interdisciplinary Center of Marine and Environmental Investigation (CIIMAR/CIMAR), Edifício do Terminal de Cruzeiros do Porto de Leixões, Av. General Norton de Matos s/n, 4050-208 Matosinhos, Portugal; dloureiro@ff.up.pt; 5 Department of Chemical Sciences, Laboratory of Organic and Pharmaceutical Chemistry, Faculty of Pharmacy, University of Porto, 4050-313 Porto, Portugal; cgoncalves.azevedo@gmail.com

**Keywords:** pyranoxanthone, antitumor, paclitaxel, mitosis, apoptosis, cancer

## Abstract

Microtubule-targeting agents (MTAs) remain a gold standard for the treatment of several cancer types. By interfering with microtubules dynamic, MTAs induce a mitotic arrest followed by cell death. This antimitotic activity of MTAs is dependent on the spindle assembly checkpoint (SAC), which monitors the integrity of the mitotic spindle and proper chromosome attachments to microtubules in order to ensure accurate chromosome segregation and timely anaphase onset. However, the cytotoxic activity of MTAs is restrained by drug resistance and/or toxicities, and had motivated the search for new compounds and/or alternative therapeutic strategies. Here, we describe the synthesis and mechanism of action of the xanthone derivative pyranoxanthone **2** that exhibits a potent anti-growth activity against cancer cells. We found that cancer cells treated with the pyranoxanthone **2** exhibited persistent defects in chromosome congression during mitosis that were not corrected over time, which induced a prolonged SAC-dependent mitotic arrest followed by massive apoptosis. Importantly, pyranoxanthone **2** was able to potentiate apoptosis of cancer cells treated with nanomolar concentrations of paclitaxel. Our data identified the potential of the pyranoxanthone **2** as a new potent antimitotic with promising antitumor potential, either alone or in combination regimens.

## 1. Introduction

Cancer remains a major cause of death worldwide and new cases resulting from an aging population are expected to increase [1]. Remarkable progress has been made to fight cancer in recent decades, namely in the field of immunotherapy. For example, patients with more than a dozen types of cancer have received immunotherapies approved by the United States Food and Drug Administration (FDA) over the past five years, with a significant drop in the death rate [2,3,4,5]. Despite this progress, other anticancer strategies are needed because many cancer patients still do not respond to immunotherapy. The biological transformation of a normal cell into a cancer cell depends on several environmental and/or genetic factors [6]. Nonetheless, the growth and proliferation of normal or tumor cells is invariably dependent on mitosis, the process that originates two daughter cells from a parental cell. Mitosis plays a central role during the multi-step tumorigenic process due to the high rate of cell proliferation [7]. Under normal conditions, mitosis is strikingly monitored by the spindle assembly checkpoint (SAC) mechanism to ensure the fidelity of chromosome segregation at the metaphase to anaphase transition [4]. However, when the SAC activity is deficient, this can lead to aneuploidy, one of the cancer hallmarks [7,8,9].

The SAC is a highly sensitive mechanism responsible for monitoring, at kinetochores, the attachment of chromosomes and spindle microtubules during mitosis [8]. At unattached and/or tensionless kinetochores, the SAC is activated and halts metaphase to anaphase transition by generation of the mitotic checkpoint complex (MCC). The MCC results from the association of SAC proteins Mad2 (mitotic arrest deficiency 2), Bub3 (budding uninhibited by benomyl 3), Bub1-related1 (BubR1), and Cdc20 (cell-division cycle protein 20) to inhibit the anaphase-promoting complex/cyclosome (APC/C), thereby preventing premature mitotic exit [8]. Once all kinetochores are attached to spindle microtubules and under tension, and all chromosomes are aligned at metaphase plate, the SAC is silenced, promoting mitosis exit [10,11]. Thus, SAC ensures timely anaphase onset and prevents the generation of aneuploid cells [12].

Taxanes (e.g., paclitaxel) and vinca alkaloids (e.g., vinblastine) are front-line chemotherapeutics in clinical practice for several cancer types [13]. By disturbing microtubule dynamics, these microtubule targeting agents (MTAs) mainly induce a SAC-dependent mitotic arrest, followed by apoptotic cell death, promoting the elimination of cancer cells [12]. However, the variability of patients’ response to the treatment, and resistance and toxicity issues associated with MTAs boosted the search for new microtubules poisons or new therapeutic strategies.

**Figure 1:**
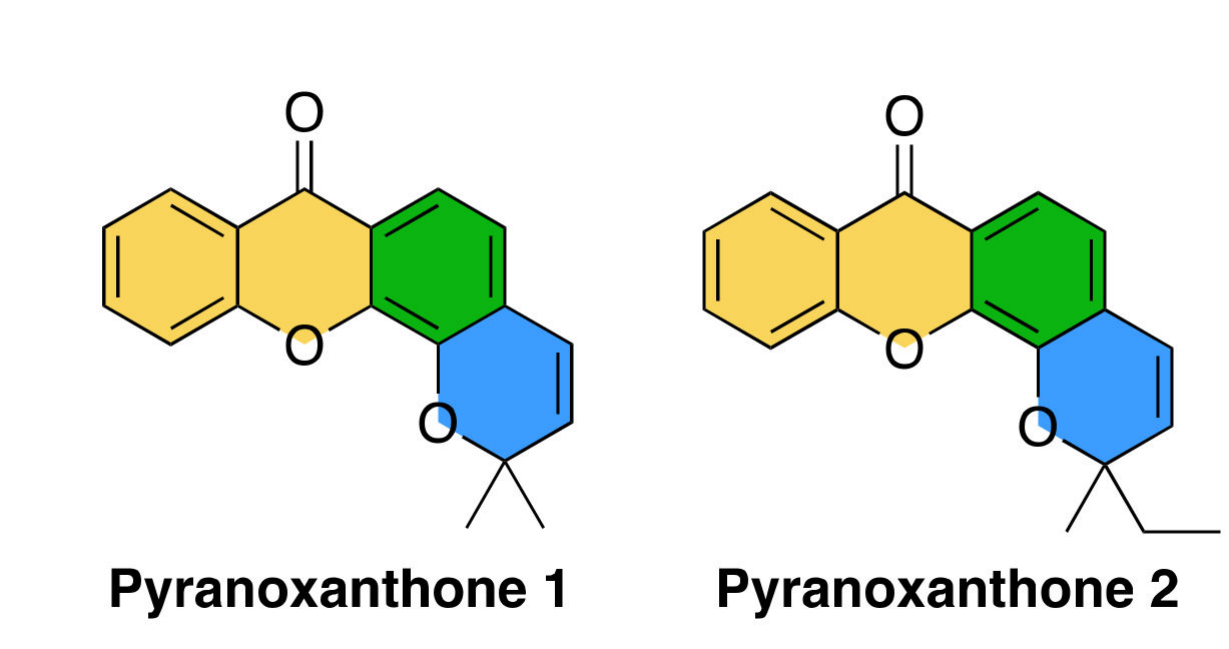
Structure of pyranoxanthone **1** and **2**. The xanthone scaffold is highlighted in yellow and green and the benzopyran scaffold is highlighted in green and blue.

Xanthones and benzopyrans are two important *O*-heterocyclic scaffolds present in natural products [14,15,16]. Considered to be “privileged structures”, these scaffolds have acquired a substantial importance in Medicinal Chemistry as depending on their substitution pattern they can show different biological activities [17,18]. Even considering only the antitumor activity, the biological activity of xanthones depends on the position and types of substituents on the scaffold providing a broad spectrum of activity [19], namely the interference with various mechanisms of the tumor cell: blocking the cell cycle, preventing the proliferation of the tumor cell, directing apoptosis, triggering DNA repair, functioning as an anti-inflammatory agent, and preventing the recruitment of cells necessary in angiogenesis and, consequently, metastasis [20]. This propelled us to design and produce synthetic pyranoxanthone derivatives, which by overcoming the biosynthetic pathways impositions allows the merging of xanthone (Figure 1, highlighted with yellow and green) and benzopyran (Figure 1, highlighted with green and blue) scaffolds [21,22]. From this endeavor, the pyranoxanthone **1** (Figure 1) was identified which exhibited a potent growth inhibitory activity against the human tumor cell lines MCF-7 (breast adenocarcinoma) and A375-C5 (melanoma) [21]. Even though its growth inhibitory mechanism is still unknow, the angular fused ring orientation without substituents appears to be important for the activity. Retaining these features, pyranoxanthone **2** was designed to assess the significance of the two alkyl groups in the chromene moiety for the antitumor activity (Figure 1). Hence, in the present study, we report the mechanism elucidation of the lead compound pyranoxanthone **2**, identified as a lead compound with potent cytotoxic activity against cancer cells, and determined its potential to increase the sensitivity of cancer cells to low doses of paclitaxel. Our data highlight the potential of the pyranoxanthone **2** as a new and promising antimitotic with effective antitumor activity in vitro.

## 2. Results

### 2.1. Synthesis of Pyranoxanthone 2

**Scheme 1:**
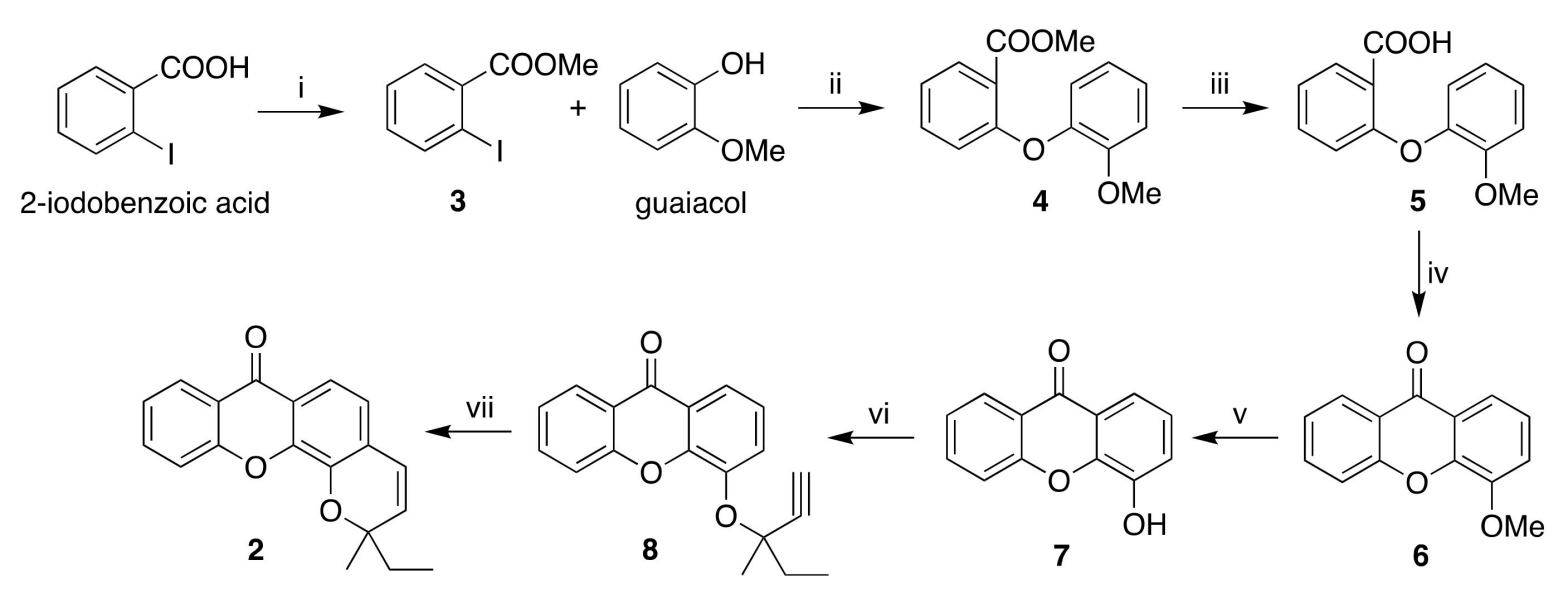
Synthesis of pyranoxanthone **2**. Reagents and conditions: (i) H_2_SO_4_, methanol, reflux, **3**: 95%; (ii) K_3_PO_4_, CuI, picolinic acid, dimethyl sulfoxide (DMSO), 90 °C, **4**: 40%; (iii) LiOH, tetrahydrofuran/methanol, room temperature (RT), **5**: 88%; (iv) CH_3_COCl, H_2_SO_4_, RT, **6**: 85%; (v) AlCl_3_, toluene, reflux, **7**: 79%; (vi) 3-methylpent-1-yn-3-ol, trifluoroacetic anhydride (TFAA), 1,8-diazabicyclo[5.4.0]undec-7-ene (DBU), CuCl_2_, acetonitrile, −5 °C, **8**: 67%; (vii) *N*,*N*-dimethylformamide (DMF), microwave (MW), 150 °C, **2**: 86%.

The synthetic pathway to pyranoxanthone **2** is similar to the one previously described by us [21]. The pathway involved the *O*-propargylation of 4-hydroxyxanthone (**7**) followed by cyclization to the target compound (Scheme 1).

4-Hydroxyxanthone (**7**) is a crucial precursor of both pyranoxanthone **1** and **2** and its synthesis is based on diaryl ether key intermediate, which is usually obtained by a copper-catalyzed Ullmann ether reaction [21]. α-Amino acids such as *N*,*N*-dimethylglycine, have been used as ligands to promote the Ullmann coupling reaction [23]. However, in our previous work diaryl ether intermediate was obtained in poor yields using *N*,*N*-dimethylglycine as co-catalyst [21]. To optimize this step, picolinic acid was used as co-catalyst in a different base-solvent system [24]. In fact, the coupling building block **3**, obtained by esterification of 2-iodobenzoic acid, with guaiacol yielded intermediate **4** with a better yield of (40% vs 15% [21]). Hydrolysis of methyl ester **4** yielded the intermediate **5**, which was cyclized to the xanthone derivative **6** by an acid-catalyzed intramolecular electrophilic cycloacylation. Ether cleavage of xanthone derivative **6** yielded 4-hydroxyxanthone **7**. 4-hydroxyxanthone **7** was *O*-propargylated with a trifluoroacetate, formed in situ by the reaction of 3-methylpent-1-yn-3-ol with trifluoroacetic anhydride (TFAA), yielding the aryl ethylmethylpropargyl ether **8**, which was cyclized to yield pyranoxanthone **2** by microwave(MW)-assisted thermal rearrangement (supplementary Figures S1–S4).

### 2.2. The Pyranoxanthone 2 Has a Potent Growth Inhibitory Activity Against Tumor Cells

To test the ability of pyranoxanthone **2** to inhibit in vitro tumor cell growth, we performed a Sulforhodamine B (SRB) assay and determined the GI_50_ of the compound, corresponding to the concentration that was able to cause 50% cell growth inhibition. Forty-eight hours after treatment, the compound pyranoxanthone **2** showed potent growth inhibitory activity against the three human cancer cell lines tested: melanoma (A375-C5), breast (MCF-7) and lung (NCI-H460) with a GI_50_ value of 5.29 ± 1.19, 6.61 ± 0.78 and 6.67 ± 0.20 μM, respectively. This result supports the potential of pyranoxanthone **2** as promising anticancer agent and prompt us to explore their cytotoxic mechanism.

### 2.3. The Pyranoxanthone 2 Induces Mitotic Arrest of Cancer Cells

**Figure 2:**
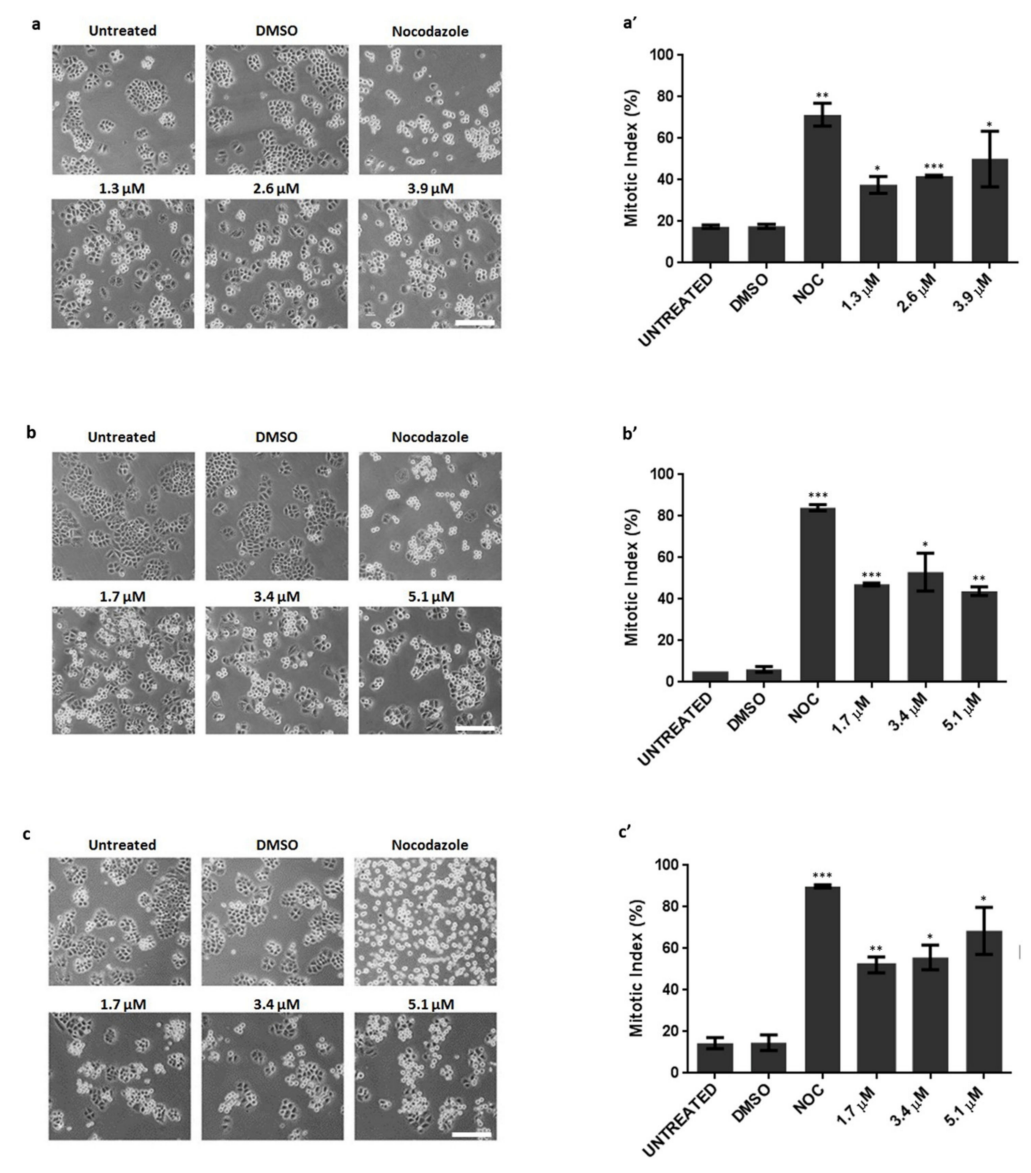
Cancer cells arrested in mitosis, in response to pyranoxanthone 2 (PX2) treatment. Left-Representative phase contrast microscopy images, after 16 h treatment with PX2, at indicated concentrations, displaying accumulation of mitotic cells (rounded) in (**a**) A375-C5, (**b**) MCF-7 and (**c**) NCI-H460 cancer cell lines. Nocodazole was used as a positive control. DMSO was used as a compound solvent control. Bar, 10 µm. Right-Mitotic index graphs showing accumulation of mitotic cells in (**a’**) A375-C5, (**b’**) MCF-7 and (**c’**) NCI-H460 cancer cells with statistical relevance of * *p* < 0.05, ** *p* < 0.01 and *** *p* < 0.001 by unpaired *t*-test.

One possible explanation for the anti-growth activity is that pyranoxanthone **2** interferes with cell division. Thus, we evaluated whether the compound pyranoxanthone **2** presents antimitotic activity. Cells were treated with pyranoxanthone **2** and observed by phase contrast microscopy. Effectively, 16 h post-incubation with pyranoxanthone **2**, we noticed a significant increase in rounded and bright cells reminiscent of cells arrested in mitosis. This accumulation in mitosis was observed in the three cancer cell lines tested, comparatively to untreated or dimethyl sulfoxide (DMSO)-treated cells (Figure 2a–c), and further confirmed by the increase in the mitotic index (Figure 2a’–c’). As expected, Nocodazole, included here as a positive control, induced a significant accumulation of mitotic cells from mitotic. Thus, pyranoxanthone **2** exerts its anti-growth activity by acting as an antimitotic agent.

### 2.4. Treatment with Pyranoxanthone 2 Leads to Chromosome Congression Defects

**Figure 3:**
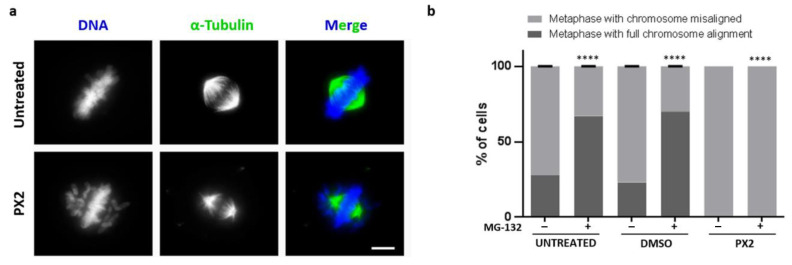
Pyranoxanthone 2 (PX2) treatment results in chromosome misalignment phenotype. (**a**) Immunofluorescence images of an untreated cell in metaphase with all chromosomes aligned at equatorial zone (Top), and a PX2-treated cell, arrested in prometaphase-like state, showing many misaligned chromosomes (Bottom). Microtubules (green) were stained with anti-α-tubulin antibody and DNA (blue) with 4’,6’-diamino-2-fenil-indol (DAPI). Bar, 5 µm. (**b**) Graphical representation of data shown in (**a**), in absence (−) or presence (+) of proteasome inhibitor MG-132 with statistical relevance of **** *p* < 0.0001 by two-way ANOVA with Tukey’s multiple comparisons test.

To gain insight into the mechanism behind the mitotic arrest induced by the compound pyranoxanthone **2**, we performed an immunofluorescence assay to visualize DNA and mitotic spindle morphology. Herein, and for further studies, the NCI-H460 cell line was selected since this cell line presented the highest mitotic index with the lower concentration of pyranoxanthone **2** used, and also due to its suitability for quantitative evaluation of morphological changes. By fluorescence microscopy analysis, we confirmed the mitotic arrest by the presence of the mitotic spindle and DNA condensation (Figure 3a). Interestingly, we observed that the majority of pyranoxanthone **2**-treated cells showed several misaligned chromosomes, contrary to untreated cells in which all chromosomes were aligned at the metaphase plate (Figure 3a). Also, the density of the mitotic spindle seems to be affected by the treatment, with seemingly less microtubule fibers than in untreated cells where a robust bipolar mitotic spindle was evident (Figure 3a). We then proceeded to the quantification of the number of full metaphases and metaphases with misaligned chromosomes, in untreated and pyranoxanthone **2**-treated cells, in the presence or absence of the proteasome inhibitor MG-132. By inhibiting the proteasome degradation of mitotic substrates such as securin and cyclin B, MG-132 blocks the transition from metaphase to anaphase. Thus, we used MG-132 to extend the duration of mitosis in pyranoxanthone **2**-treated cells in order to provide more time for the cells to correct and align all chromosomes at the metaphase plate [22]. In control cells, the percentage of metaphases with all chromosomes aligned significantly increased after addition of MG-132 (Figure 3b). However, in pyranoxanthone **2**-treated cells, 100% of metaphases still exhibited several misaligned chromosomes even after addition of MG-132 (Figure 3b). This analysis indicates that pyranoxanthone **2** induces persistent and uncorrectable and irreversible defects in chromosome congression.

### 2.5. Treatment with Pyranoxanthone 2 Interferes with Kinetochore-Microtubules Attachments Stability

**Figure 4:**
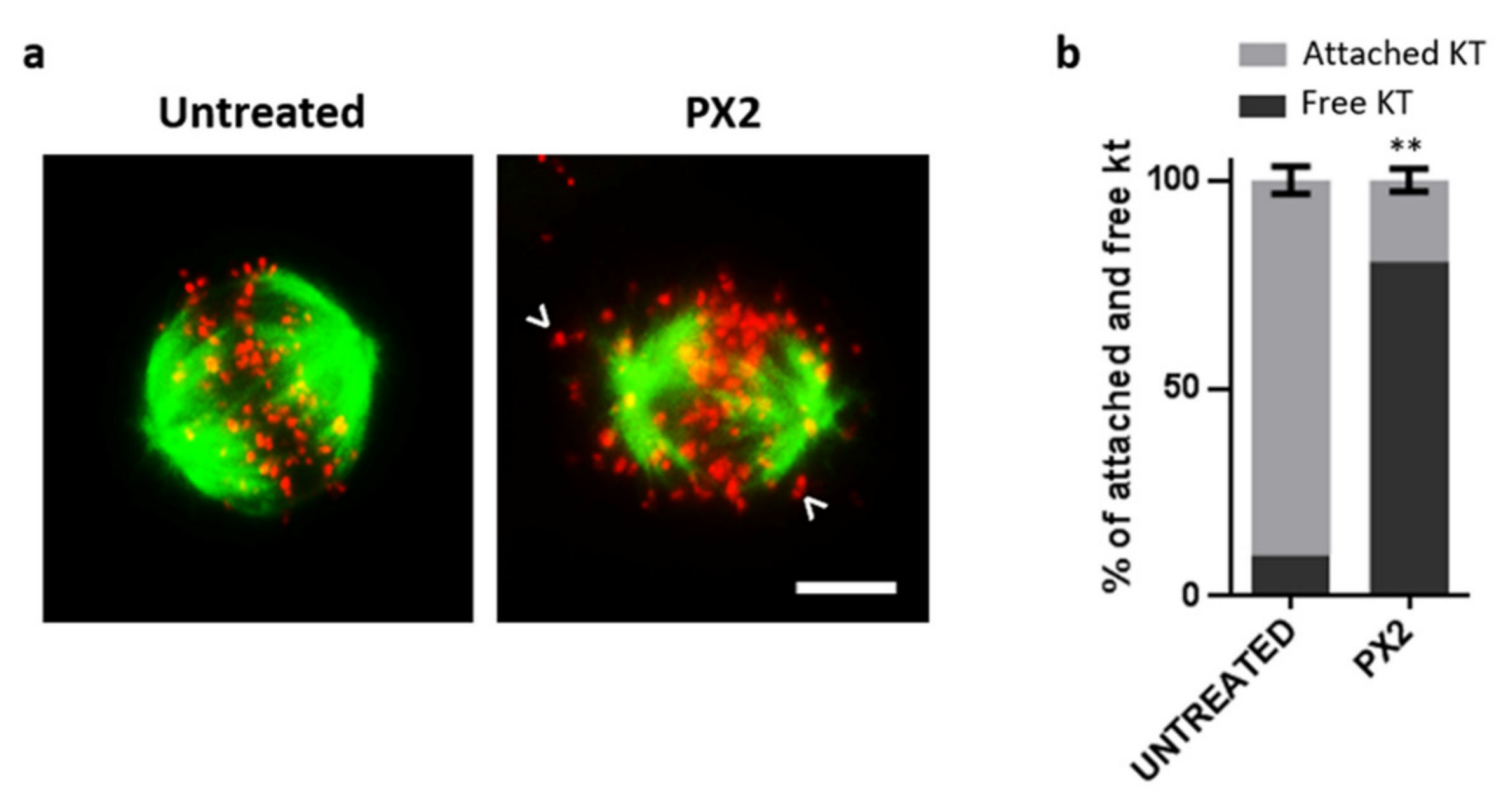
Pyranoxanthone 2 (PX2) treatment generates loss of stability of kinetochore-microtubule attachments. (**a**) Representative immunofluorescence images after cold treatment assay, showing several unattached kinetochores (free red spots pointed by the white arrowheads) in cells with PX2 treatment, whereas most kinetochores were attached (Red spots with attached green fibers) in untreated cells. Microtubules (green) were stained with anti-α-tubulin antibody, kinetochores (red) with anti-CREST antibody, and DNA (blue) with DAPI. (**b**) Quantification of cold-stable microtubules (as percentage of attached kinetochores per cell) after treatment with PX2 with statistical relevance of ** *p* < 0.01 by unpaired *t*-test.

The induction of a persistent misalignment chromosome phenotype suggests that pyranoxanthone **2** weakens kinetochore-microtubule (KT-MT) attachments [12], as robust KT-MT are required for accurate chromosome congression and mitosis progression. To test this hypothesis, we performed a functional assay termed cold treatment. By subjecting cells to low temperature for a short period, it is possible to distinguish between robust and weak KT-MT attachments: under low temperature, only stable and functional kinetochore-attached microtubules (or K-fibers) are still present, while weakly attached microtubules are disassembled. Immunofluorescence assay was performed using an anti-CREST (Raynaud’s phenomenon, esophageal dysmotility, sclerodactyly, and telangiectasias) antibody to localize kinetochores, and an anti-α-tubulin antibody to visualize the spindle microtubules. We found that pyranoxanthone **2**-treated cells showed few cold-resistant K-fibers and several free kinetochores. Instead, in untreated cells, all kinetochores were attached to microtubules (Figure 4a,b). The data indicates that the compound pyranoxanthone **2** weakens KT-MT attachments thereby compromising chromosome movement and alignment, which may explain the congression defects described above.

### 2.6. Treatment with Pyranoxanthone 2 Elicits Spindle Assembly Checkpoint Activation

**Figure 5:**
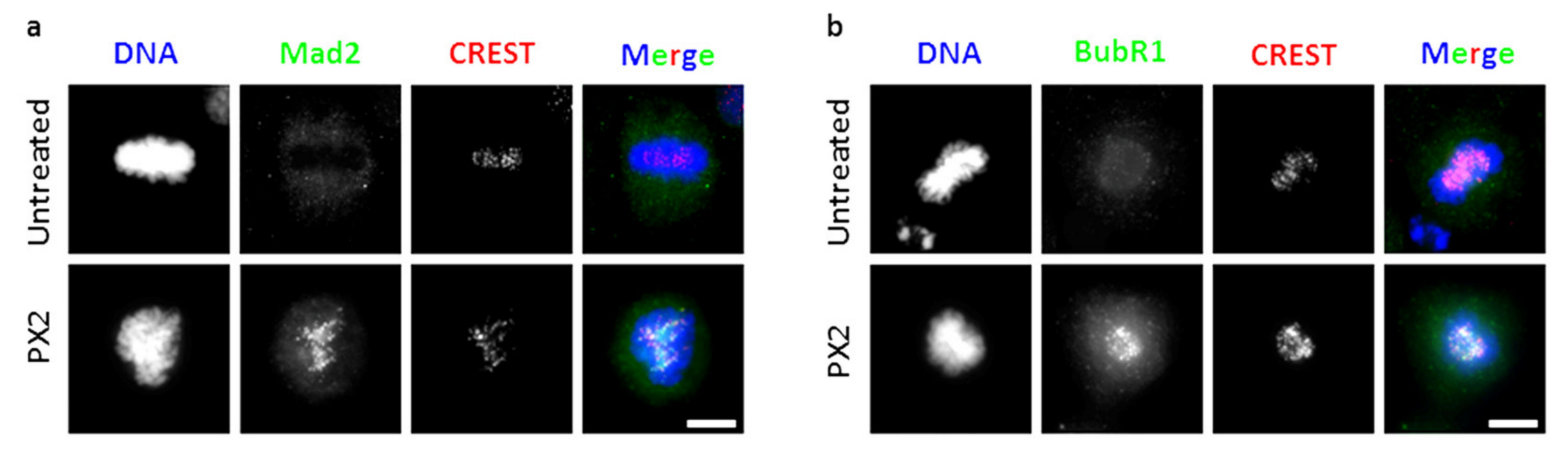
Treatment with pyranoxanthone **2** (PX2) triggers the spindle assembly checkpoint. Immunofluorescence staining using antibodies against (**a**) Mad2 (green), (**b**) BubR1 (green), CREST (red) and DAPI (blue) in untreated and PX2-treated cells, as indicated. In untreated cells, Mad2 and BubR1 is not located in kinetochores, consistent with their normal localization pattern. In PX2-treated cells (bottom panel), these proteins are present in all mitotic cells, indicating spindle assembly checkpoint activation. Bar, 5 µm.

As antimitotic agents rely on SAC activity to induce mitotic arrest, we wondered whether the antimitotic activity of pyranoxanthone **2** is also SAC-dependent. Unattached and/or improperly attached kinetochores lead to SAC activation, through assembly of the mitotic checkpoint complex (MCC). The proteins Mad2 and BubR1 are part of MCC complex, which remains at kinetochores until full KT-MT attachment and chromosome alignment at metaphase plate are achieved. Then, Mad2 and BubR1 leaves the kinetochores to promotes MCC disassembly and SAC silencing. Thus, the presence of Mad2 and BubR1 at kinetochores has been used as markers of SAC activity [22]. To evaluate the SAC activity in pyranoxanthone **2**-treated cells, we immunostained treated cells with antibodies against Mad2 and BubR1. An anti-CREST antibody was used to localize the kinetochores (Figure 5a,b). From fluorescence microscopy analysis, Mad2 and BubR1 were undetectable at kinetochores of untreated metaphase cells, indicative of SAC inactivation (Figure 5a,b). In pyranoxanthone **2**-treated cells, strong Mad2 and BubR1 staining was observed at metaphase kinetochores (Figure 5a,b), consistent with chronic SAC activation. Therefore, the mitotic arrest induced by the pyranoxanthone **2** is sustained by a chronically active SAC due to persistent misattached chromosomes.

### 2.7. Cell Fates of Cancer Cells Arrested in Mitosis by the Pyranoxanthone 2

**Figure 6:**
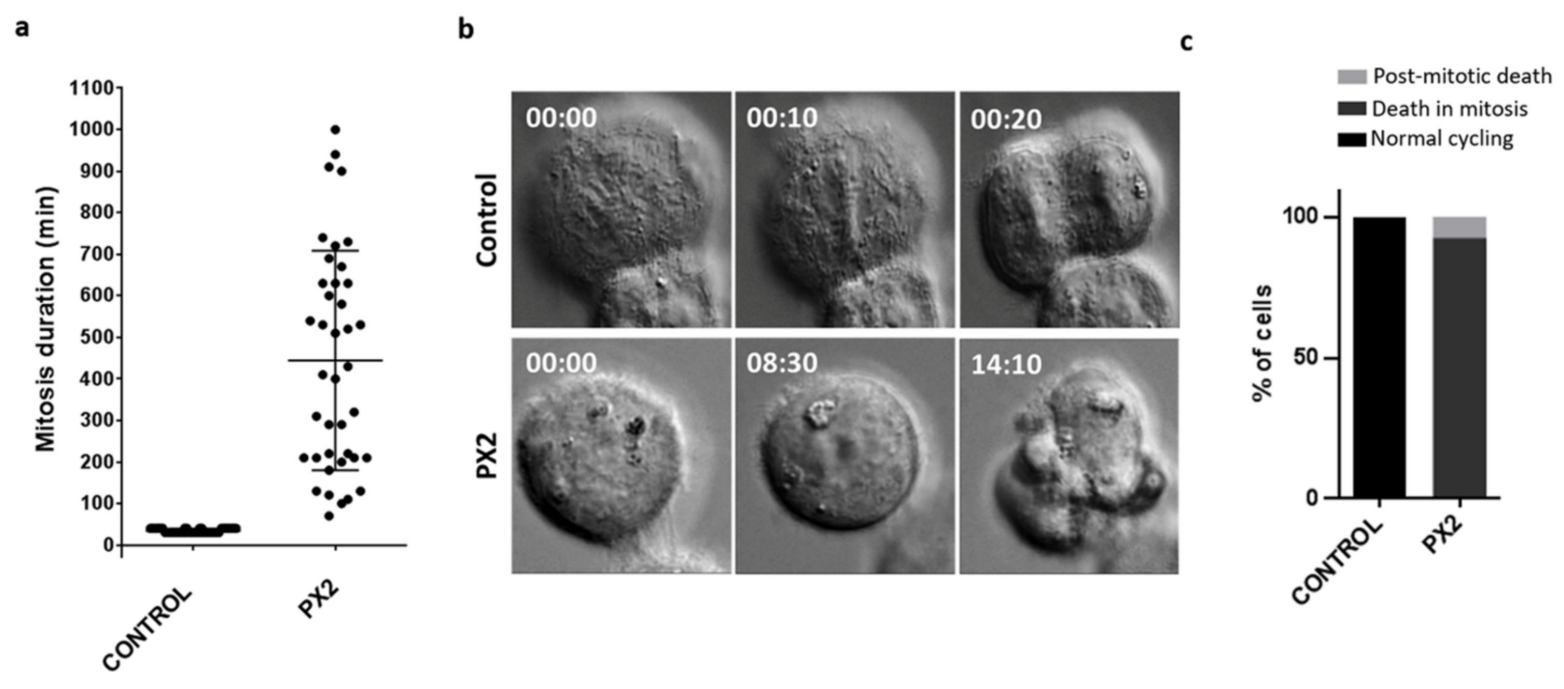
Pyranoxanthone 2 (PX2) treatment promotes death in mitosis, after prolonged arrest. (**a**) Mitosis duration as determined by time-lapse microscopy, in untreated (control) and PX2-treated cells. Each spot represents one cell. (**b**) Representative time-lapse sequences of untreated and PX2-treated cells. Untreated cell undertakes mitosis for about 30 min (top), while PX2-treated cell (bottom) arrests in mitosis during several hours (457.8 ± 264.2 min) followed by death. Numbers indicate times in 00 h:00 min. Movies are available as Supplementary materials. (**c**) Quantification of cell fate after PX2 treatment. The percentage of cells undergoing post-mitotic death (PMD) and death in mitosis (DIM), and cells with normal cycling, over the total number of cells are represented.

To further understand the mechanistic underlying the cytotoxic activity of the pyranoxanthone **2**, we analyzed the cell fate of mitosis-arrested cells by live-cell imaging using time lapse differential interference contrast (DIC) microscopy, over 48 h time course. First, we found that pyranoxanthone **2**-treated cells (*n* = 40) lasted in mitosis (from nuclear envelope breakdown to anaphase onset) 457.8 ± 264.2 min on average, more than 16-fold when compared to the duration of mitosis in untreated cells (28 ± 6.64 min) (Figure 6 and supplementary video S1 and supplementary video S2). Then, survival fate analysis of each mitotic cell revealed that 92.5% of pyranoxanthone **2**-treated cells died in mitosis, and 7.5% of cells underwent post-mitotic death (Figure 6 and videos S1 and S2).

**Figure 7:**
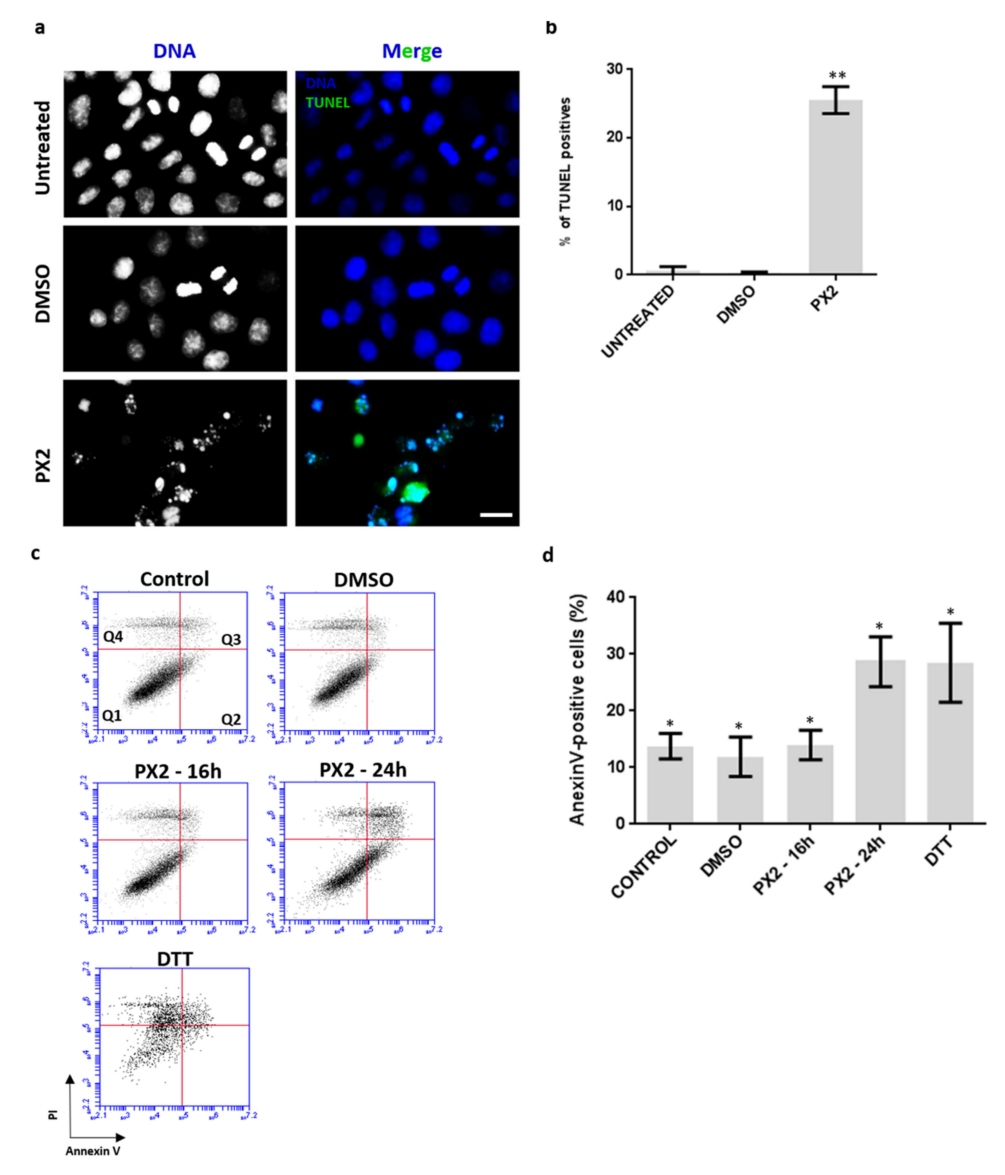
Prolonged exposure to pyranoxanthone 2 (PX2) leads to apoptotic cell death. (**a**) TUNEL staining showing accumulation of apoptotic cells (green) in cells treated with the compound for 48 h. DNA was counterstained with DAPI (blue). Bar, 5 µm. (**b**) Apoptotic index in control cells and upon 48 h compound treatment, expressed as a percentage of total cells with statistical relevance of ** *p* < 0.01 by unpaired *t*-test. (**c**,**d**) Flow cytometry analysis of apoptosis by Annexin V/PI co-staining, 16 and 24 h after PX2 treatment. (**c**) Representative cytogram and (**d**) quantification of Annexin V-positive cells are shown with statistical relevance of * *p* < 0.05 by unpaired *t*-test. The quadrants Q were defined as Q1 = live (Annexin V- and PI-negative), Q2 = early stage of apoptosis (Annexin V-positive/PI-negative), Q3 = late stage of apoptosis (Annexin V- and PI-positive) and Q4 = necrosis (Annexin V-negative/PI-positive).

During the aforementioned immunofluorescence assays, we noticed that some cells exhibited abnormal nuclear morphology, suggestive of cell death by apoptosis. To verify this possibility, we performed TUNEL and Annexin V/PI assays, after 24 h of pyranoxanthone **2** treatment. The percentage of TUNEL-positive cells among cells treated with pyranoxanthone **2** was 25.53 ± 1.97%, while in untreated and DMSO-treated cells it was 0.60 ± 0.50% and 0.27 ± 0.12%, respectively (Figure 7a,b). From flow cytometry analysis, after annexin V/PI labeling, the percentage of apoptosis was 13.76 ± 2.24%, and 11.89 ± 3.49% in untreated and DMSO-treated cells, respectively, while it reached 28.67 ± 3.52% after 24 h treatment with pyranoxanthone **2**, very similar to that obtained after treatment with 1,4-dithiothreitol (DTT) used here as a positive control (Figure 7c,d). Altogether, these results clearly indicate that pyranoxanthone **2** exerts its cytotoxic activity by inducing apoptosis after a prolonged mitotic arrest.

### 2.8. Treatment with Pyranoxanthone 2 Enhances Paclitaxel Cytotoxicity

**Figure 8:**
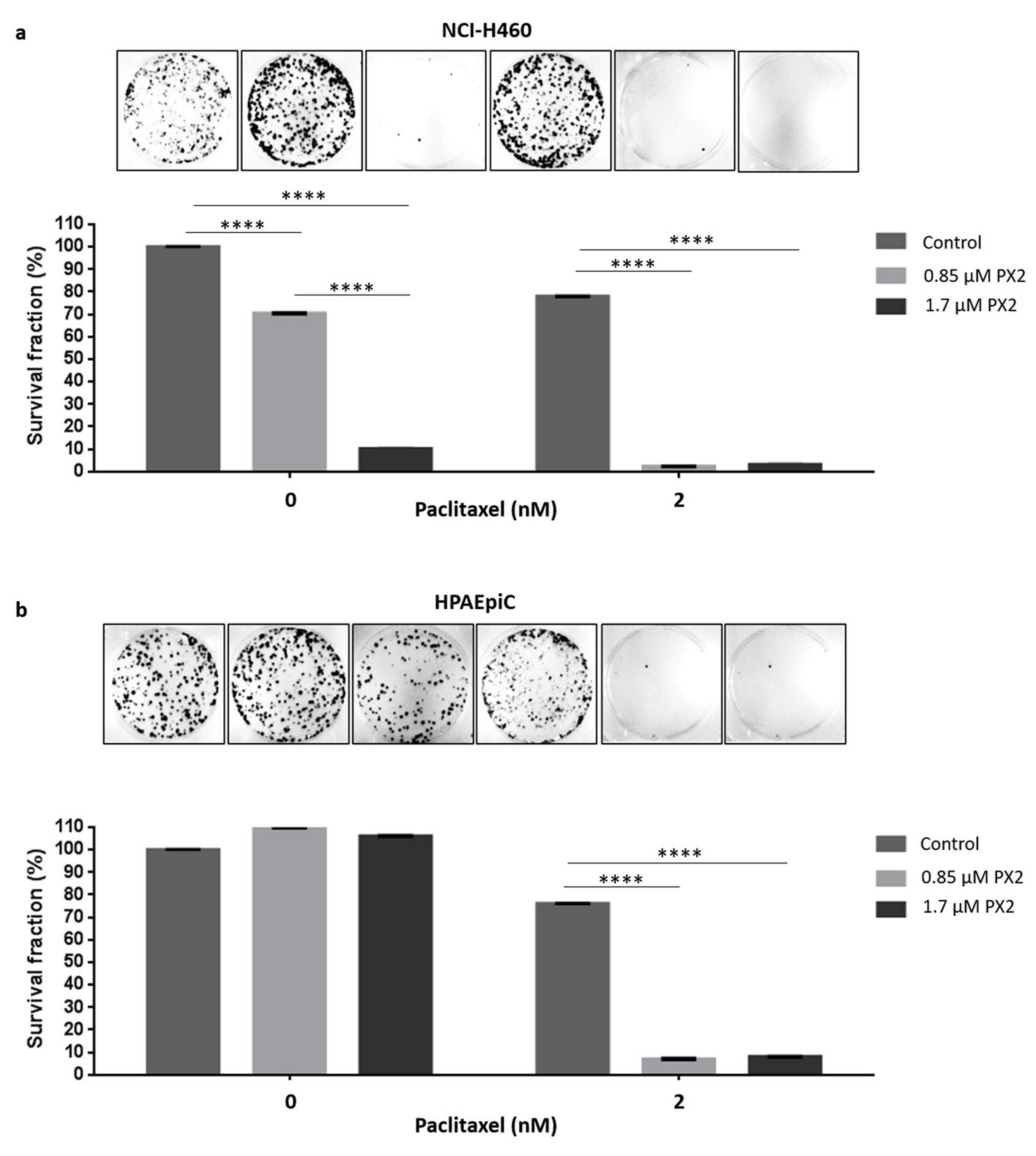
Pyranoxanthone 2 (PX2) treatment enhances paclitaxel-mediated cytotoxicity. NCIH460 (**a**) HPAEpiC (**b**) cells were treated with 0.85 μM and 1.7 μM of PX2 and/or paclitaxel (PTX) at 2 nM, for 48 h. A 8-days colony-formation assay was performed. After washout, the surviving colonies were stained with crystal violet and a representative figure is shown for each condition. Quantification of data shown was performed and the results are the mean from three independent experiments, expressed as % of survival fraction. Statistical relevance of **** *p* < 0.0001 by two-way ANOVA with Tukey’s multiple comparisons test was used.

Taking into account the potent cytotoxic activity of pyranoxanthone **2** and given the limitations of current paclitaxel-based chemotherapy, we explored the potential of pyranoxanthone **2** to increase the sensitivity of cancer cells to clinically relevant concentrations of paclitaxel. To address this question, we performed a long-term proliferation assay. Cells were treated with 0.85 µM of pyranoxanthone **2** (almost 8-fold less GI_50_), alone or in combination with 2 nM of paclitaxel, for 48h. Eight-days later, treatment with 0.85 µM of pyranoxanthone **2** had no effect on the ability of cancer cells to proliferate and form colonies, behaving as untreated cells (Figure 8a). Interestingly, when pyranoxanthone **2** was added prior paclitaxel treatment, the inhibitory effect on cancer cell proliferation was significantly higher than that observed in cells treated with pyranoxanthone **2** or paclitaxel alone (Figure 8a). Importantly, at the highest concentration (1.7 µM) that killed more than 90% of tumor cells, the compound does not affect the survival of the lung non-tumor HPAEpiC cells indicating that the compound is toxic to tumor cells but not to non-tumor cells (Figure 8b). One possible explanation for this observation is that non-tumor cells have a lower proliferative rate than cancer cells, being less affected by pyranoxanthone 2. Indeed, the mitotic index of HPAEpiC cells was <12% at the highest concentration of pyranoxanthone 2 (5.1 µM) used (Supplementary Figure S5), much lower than >50% achieved by cancer cells at the same concentration (Figure 2). Similarly, HPAEpiC cells exhibited lower mitotic index (up to 25%) than cancer cells (up to 85%), when treated with nocodazole, used as a positive control of antimitotic drugs. This suggests that pyranoxanthone **2** treatment sensitizes cancer cells to cytotoxic effect of paclitaxel. Of note, the concentrations of both agents in combination regimens could be significantly lowered.

## 3. Discussion

Despite the widespread clinical use of antimitotic drugs, namely drugs that target microtubules such as paclitaxel, the intrinsic and/or acquired resistance and the associated toxicities remain major problems to be overcome to fight cancer [25]. This issue has motivated the intensive search for new compounds and/or new therapeutic strategies for cancer treatment. In this field, several natural products have been evaluated as potential therapeutic agents. Increasing attention has been paid to xanthone derivatives, from natural sources and synthetic routes, to discover new drug candidates with, among others, anticancer properties [19].

In the present study, we unveiled the mechanism of the cytotoxic activity of the xanthone derivative pyranoxanthone 2. We demonstrated that pyranoxanthone 2 disturbs the mitotic spindle, which lead to unstable kinetochore-microtubules attachments and chromosome misalignment that activate SAC, thereby leading to prolonged mitotic arrest and, subsequently, cell death, in NCI-H460 human lung carcinoma. To ruled out a cell line specific phenotype, we also analyzed the effect on chromosome alignment and on cell death in A375-C5 melanoma and MCF-7 breast cancer cell lines. We showed that after incubation with PX2, both studied cell lines also exhibited misaligned chromosomes, as shown by immunofluorescence analysis, indicative of instable chromosome attachment and, thus, of disturbed microtubules of the mitotic spindles. These treated cells also undergo apoptosis as shown by flow cytometry analysis 48h after PX2 treatment (supplementary Figure S6). Overall, the data indicate that the compound exerts its cytotoxic activity in different cancer cell types with similar mechanistic.

Unstable attachments were reported to lead to chromosome missegregation which, depending on their severity, culminate in the generation of aneuploidy cells or cell death [23]. Therefore, the misalignment phenotype exhibited under pyranoxanthone 2 treatment seems to be massive enough to be incompatible with cell viability. Importantly, this misalignment was found in almost 100% of the pyranoxanthone 2-treated cells underwent death in mitosis. This is very relevant, since after a mitotic arrest cells can exit from mitosis without cell division, in a process called mitotic slippage [9,23], and escape from death contributing to cancer resistance. Such possibility does not happen in cells treated with pyranoxanthone 2.

Interestingly, when we tested nanomolar concentrations of pyranoxanthone 2 in co-treatment with clinically relevant concentrations of paclitaxel, in a long-term colony formation assay, we found an enhancement of the anti-proliferative activity. This finding is probably due: (i) to the mitotic arrest induced by pyranoxanthone 2 treatment which delays cell cycle progression giving time to paclitaxel in order to exert their cytotoxic activity; and/or (ii) to a potentiation of chromosome segregation errors resulting from the additive effect of pyranoxanthone 2 and paclitaxel. Indeed, both pyranoxanthone 2 and paclitaxel at nanomolar concentrations (<10 nM) promote missegregated chromosomes and aneuploidy [26,27].

Importantly, enhancing chromosome missegregation and aneuploidy was previously reported as a possible strategy to sensitize cancer cells to paclitaxel and docetaxel [28,29].

Moreover, lowering the concentrations of both paclitaxel and pyranoxanthone 2 is expected to minimize cytotoxicity and side effects. Xanthone derivatives have also exhibited therapeutic potential in combinatorial therapeutic modalities. For instance, 5,6-dimethylxanthone-4-acetic acid (DMXAA) exhibited either alone or in combination in vitro and in vivo anticancer activity in non-small cell lung cancer by the regulation of proteins involved in signaling pathways, such as cell cycle progression and apoptosis [30]. Although DMXAA alone did not show a striking anti-tumor activity in patients, pre-clinical results showed that a co-administration of DMXAA with other drugs has an increase in anti-tumor activity, through activation of immune system by tumor necrosis factor alpha (TNF-α) induction [31]. In fact, co-treatment regimens constitute an appealing strategy to kill cancer cells more effectively than individual treatments, namely with chemotherapeutics [12].

In conclusion, the present data elucidates the mechanistic underlying the xanthone derivative pyranoxanthone 2, identifying it as a potent antimitotic agent with promising potential as anticancer drug, either alone or in combinatorial regimens. Future in vivo studies are needed to further explore the anticancer potential of pyranoxanthone 2.

## 4. Materials and Methods

### 4.1. Chemistry

#### 4.1.1. General Methods

All reagents and solvents were purchased from Sigma-Aldrich (Sigma-Aldrich Co. Ltd., Gillingham, UK) or Alfa Aesar (Thermo Fisher GmbH, Kandel, Germany) and were used without further purification. Solvents were evaporated using a Rotavapor^®^ R-300 (Büchi, Flawil, Switzerland). Thin-layer chromatography carried out on Merck silica (GF254, 0.2 mm thickness) precoated plates and purifications were performed by chromatography flash column using Merck silica gel 60 (0.040–0.063 mm, Merck, Darmstadt, Germany). MW reactions were carried out on an Ethos MicroSYNTH 1600 Microwave Labstation (Milestone, Sorisole, Italy). Melting points (mp) were measured in a Köfler microscope (Wagner and Munz, Munich, Germany) and were uncorrected. Fourier-transform infrared (FTIR) spectra were measured on a Nicolet iS10 FTIR spectrophotometer (software: OMNIC v. 8.3) in KBr microplates (Thermo Scientific, Waltham, MA, USA). GC-MS spectra were recorded on a Trace GC 2000 Series chromatographer coupled to GCQPlus Finnigan (Thermo Finnigan, San Francisco, CA, USA). High resolution mass spectrometry (HRMS) was performed with electrospray ionization (ESI) on a LTQ Orbitrap XL hybrid mass spectrometer (Thermo Fischer Scientific, Bremen, Germany) at CEMUP, University of Porto, Portugal. ^1^H- and ^13^C-Nuclear magnetic resonance (NMR) spectra were taken in CDCl_3_ (Deutero GmbH, Kastellaun, Germany) at room temperature on Bruker Avance 300 instrument (300.13 MHz for ^1^H and 75.47 MHz for ^13^C, Bruker Biosciences Corporation, Billerica, MA, USA). Chemical shifts relative to tetramethylsilane are expressed in δ (ppm) and coupling constants are reported in hertz (Hz). ^13^C-NMR assignments were made by 2D heteronuclear single quantum coherence and heteronuclear multiple bond correlation experiments. Compounds **3**, **5**, **6**, **7** were synthesized in our laboratory as previously described [19].

#### 4.1.2. Synthesis of Methyl 2-(2-Methoxyphenoxy)Benzoate (4)

In an oven dried flask with a magnetic stirbar, copper(I) iodide (14.5 mg, 76.3 µmol), picolinic acid (18.8 mg, 152.6 µmol), 2-iodobenzoic acid (200.0 mg, 763.2 µmol) and K_3_PO_4_ (324.0 mg, 1.5 mmol) were added. The flask was then evacuated and backfilled with nitrogen. Under a counterflow of nitrogen, 2-methoxyphenol followed by DMSO (3.0 mL) were added by syringe. The reaction mixture was heated at 90 °C for 24 h. Then, the reaction mixture was cooled to room temperature, filtered, washed with acetone, and concentrated. The product was extracted with ethyl acetate and the organic layer was washed with brine, dried over anhydrous Na_2_SO_4_, filtered and the solvent evaporated. The crude product was purified by silica gel flash chromatography (*n*-hexane/ethyl acetate/formic acid 9:1 to 7:3). Methyl 2-(2 methoxyphenoxy)benzoate (**4**) was isolated as white-yellow oil (78.9 mg, 40%).

#### 4.1.3. Synthesis of 4-((3-Methylpent-1-yn-3-yl)oxy)-9H-Xanthen-9-one (8)

To a solution of 3-methylpent-1-yn-3-ol (117.5 µL, 1.04 mmol) in anhydrous CH_3_CN under nitrogen, cooled in a salt ice-bath (−5 °C) was added 1,8-diazabicyclo[5.4.0]undec-7-ene (DBU) (250.0 µL, 1.67 mmol) followed by TFAA dropwise (152.8 µL, 1.08 mmol). The reaction mixture was stirred for 30 min. In another a two-necked flask, 4-hydroxyxanthone (200 mg, 0.942 mmol) was solubilized in anhydrous CH_3_CN and placed under nitrogen at −5 °C. To this solution was added DBU (172.9 µL, 1.15 mmol) followed by anhydrous CuCl_2_ (1.27 mg, 0.0094 mmol). To the latter reaction mixture was added dropwise via syringe to the first one, and the final reaction mixture was stirred at −5 °C for 7 h. The reaction mixture was warmed to room temperature and partitioned between water and diethyl ether. The aqueous solution was extracted with diethyl ether and the organic phase was washed with HCl 5% solution, with NaOH, and with brine. Organic layer was dried over anhydrous Na_2_SO_4_, filtered and the organic solvent evaporated. The crude product was purified by silica gel flash chromatography (*n*-hexane to *n*-hexane/ethyl acetate 9:1). 4-((3-methylpent-1-yn-3-yl)oxy)-9H-xanthen-9-one (**8**) was isolated as white solid (190 mg, 68%).

4-((3-Methylpent-1-yn-3-yl)oxy)-9H-xanthen-9-one (**8**): white solid; mp: ≥ 350 °C (dec. 170 °C). IR (KBr): $\tilde{\nu }$ = 3441, 3296, 2974, 2924, 2851, 2109, 1661, 1603, 1487, 1443, 754 cm^−1^; ^1^H NMR (300 MHz, CDCl_3_) δ = 8.34 (dd, J = 8.0, 1.7, 1H), 8.07 (dd, J = 8.0, 1.6, 1H), 7.80 (dd, J = 7.9, 1.6, 1H), 7.73 (ddd, J = 8.7, 7.1, 1.7, 1H), 7.54 (dd, J = 8.4, 1.1, 1H), 7.39 (ddd, J = 8.1, 7.0, 1.1, 1H), 7.30 (t, J = 8.0, 1H), 2.54 (s, 1H), 2.23–1.95 (m, 2H), 1.68 (s, 3H), 1.25 (t, J = 7.4, 3H). ^13^C NMR (75 MHz, CDCl_3_) δ = 177.3, 150.1, 144.4, 134.7, 127.7, 126.7, 124.0, 123.0, 123.0, 121.6, 121.0, 118.2, 84.2, 78.9, 75.6, 35.6, 26.4, 8.9. EIMS m/z (%): 293 (15, [M + H]^+•^), 292 (18, [M]^+•^), 264 (20), 263 (100), 234 (10), 205 (16), 178 (30), 115 (19), 77 (10).

#### 4.1.4. Synthesis of 2-Ethyl-2-Methylpyrano[3,2-c]Xanthen-7(2H)-one (2)

The aryl propargyl ether (125 mg, 424.7 mmol) was solubilized in anhydrous *N*,*N*-dimethylformamide (DMF) on Teflon vessel for microwave heating under nitrogen atmosphere. The mixture was heated for 30 min at 150 °C under microwave heating. The mixture was allowed to cool to room temperature and the organic solvent was evaporated. The crude product was purified by silica gel flash chromatography (n-hexane/ethyl acetate 9,5:0,5). Pyranoxanthone (**2**) was isolated as white solid (107.4 mg, 85.9%).

5-((3-Methylpent-1-yn-3-yl)oxy)-4a,9a-dihydro-9H-xanthen-9-one (2): white solid; mp: 115–117 °C; IR (KBr): $\tilde{\nu }$ = 3455, 2969, 2918, 2845, 1659, 1606, 1464, 1449, 1330 cm^−1^. ^1^H NMR (300 MHz, CDCl_3_) δ = 8.32 (dd, J = 8.0, 1.7, 1H), 7.80 (d, J = 8.1, 1H), 7.72 (ddd, J = 8.6, 7.0, 1.7, 1H), 7.60 (dd, J = 8.5, 1.1, 1H), 7.37 (ddd, J = 8.1, 7.0, 1.2, 1H), 7.00 (d, J = 8.2, 1H), 6.47 (d, J = 9.9, 1H), 5.78 (d, J = 9.9, 1H), 1.99–1.72 (m, 2H), 1.52 (s, 3H), 1.05 (t, J = 7.4, 3H). ^13^C NMR (75 MHz, CDCl_3_) δ = 177.04, 156.16, 145.66, 141.63, 134.55, 132.60, 126.62, 125.99, 123.82, 122.61, 122.43, 121.72, 121.34, 118.39, 117.37, 80.25, 33.71, 25.91, 8.21. EIMS m/z (%): 292 (5, [M]^+•^), 277(10), 264 (15), 263 (100), 234 (6), 205 (16), 178 (13), 152 (4). HRMS (ESI): m/z calcd for C_19_H_17_O_3_ [M + H]^+^: 293.11722; found: 293.11557.

#### 4.1.5. Biological Assays

Pyranoxanthone **2** was reconstituted in sterile DMSO (Sigma-Aldrich Co. Ltd., Gillingham, UK) to a stock concentration of 60 mM. To preserve compound activity, several aliquots were prepared and stored at −20 °C. In the day of experiments, pyranoxanthone **2** was diluted in fresh culture medium at desired concentrations.

### 4.2. Cell Culture and Conditions

Human pulmonary alveolar epithelial cells HPAEpiC (a gift from Prof. Bruno Sarmento, INEB/i3S, University of Porto) were grown in DMEM medium (Dulbecco′s Modified Eagle′s, Biochrom) supplemented with 10% fetal bovine serum (FBS) and 1% non-essential amino acids (Sigma-Aldrich Co., Saint Louis, MO, USA). Melanoma A375-C5, breast adenocarcinoma MCF-7 and non-small cell lung cancer NCI-H460 (European Collection of Cell Culture, UK) cell lines were grown in Roswell Park Memorial Institute medium (RPMI-1640) (Biochrom), supplemented with 5% heat inactivated FBS (fetal bovine serum, Biochrom). All cell lines were maintained at 37 °C in a humidified incubator (Hera Cell, Heraeus) with 5% CO_2_.

### 4.3. Sulforodamine B (SRB) Colorimetric Assay

A total of 5.0 × 10^4^ cells were seeded in 96-well plates, with complete culture medium, and were maintained at 37 °C for 24h. Then, cells were treated with two-fold serial dilutions of pyranoxanthone **2**, ranging from 0 to 150 µM, for 48 h. Control groups received the same amount of DMSO, used as compound solvent, up to 0.25% concentration. Forty-eight hours later, cells were fixed with 50% (*m/v*) trichloroacetic acid (Merck Millipore, Darmstadt, Germany), for 1 h at 4 °C, washed with distilled water and stained with Sulforhodamine B (SRB, Sigma-Aldrich Co. Ltd., Gillingham, UK) for 30 min at room temperature. Stained cells were washed 5 times with 1% (*v/v*) acetic acid (Merck Millipore) and left to dry at room temperature, followed by SRB complexes solubilization through addition of 10 mM Tris buffer (Sigma-Aldrich Co. Ltd., Gillingham, UK) for 30 min. Absorbance was measured at 515 nm in a microplate reader (Biotek Synergy 2,BioTek Instruments, Inc., Winooski, VT, USA). A dose–response curve was obtained for each cell line treated with pyranoxanthone **2**, and the concentration that caused cell growth inhibition of 50% (GI_50_) was determined.

### 4.4. Mitotic Index Determination

A total of 0.15 × 10⁶ cells were seeded in a six-well plate allowing attaching for 24h. Then, cells were treated either with the compound pyranoxanthone **2**, 1 μM of Nocodazole (Sigma-Aldrich Co. Ltd., Gillingham, UK) as a positive control or 1.7μM DMSO as compound’s solvent control. Untreated cells were also included. Sixteen hours later, the mitotic index (MI) was determined by cell-rounding under phase-contrast microscopy. At least, 2000 cells were counted from random microscope fields. The MI was calculated using the following formula: MI (%) = (number of mitotic cells/total number of cells) × 100.

### 4.5. Flow Cytometry

Cells were treated as described for mitotic index determination. For cell cycle analysis, after 16 and 24 h, cells were harvested, washed in phosphate-buffered saline (PBS) twice and fixed in cold 70% ethanol at 4 °C for at least 30 min. Then, cells were treated with 5 µg/mL of propidium iodide and 100 µg/mL of RNase in PBS for 30 min and analyzed in the flow cytometer. For apoptosis detection, cells were harvested and processed with the “Annexin V-FITC Apoptosis Detection Kit” (eBioscience, Vienna, Austria) according to manufacturer’s instructions. A positive control, for cell death, with 1μM DTT was included. Data was analyzed with BD Accuri TM C6 Plus software, version 1.0.27.1 (www.bdbiosciences.com). All flow cytometry analysis was carried out using a BD Accuri™ C6 Plus Flow cytometer (BD Biosciences, Qume Drive, San Jose, CA, USA) and at least 20,000 events per sample were collected.

### 4.6. Immunofluorescence

NCI-H460 cells growing on poly-L-lysine-coated coverslips were treated with 1.7 μM of pyranoxanthone **2** for 16 h. Then, cells were fixed with fresh 2% (*w/v*) paraformaldehyde (Sigma-Aldrich Co. Ltd., Gillingham, UK) in PBS for 12 min, washed 3 times with PBS for 5 min and permeabilized with 0.5% (*v/v*) Triton X-100 diluted in PBS for 7 min. After washing in PBS, cells were blocked with 10% FBS in 0.05% Tween-20 in PBS (PBST) for 30 min at room temperature, followed by 1 h incubation with primary antibodies diluted in 5% FBS in PBST. The following primary antibodies were used: human anti-CREST (1:4000, gift from E. Bronze-da-Rocha, University of Porto, Portugal); mouse anti-α-tubulin (1:2500, Sigma-Aldrich Co. Ltd., Gillingham, UK) mouse anti-BubR1 (1:200, Milipore Chemicon), mouse anti-Mad2L1 (1:200, Sigma-Aldrich Co. Ltd., Gillingham, UK). After washing in PBST, cells were incubated for 1 h with Alexa Fluor 488 and 568 conjugated secondary antibodies (Molecular Probes, Eugene, OR, USA), diluted at 1:1500. DNA was stained with 2µg/mL 4′,6-diamidino-2-phenylindole (DAPI, Sigma-Aldrich Co. Ltd., Gillingham, UK) diluted in Vectashield mounting medium (Vector, H-1000, Burlingame, CA, USA).

### 4.7. Functional Assays for Kinetochore-Microtubule (KT-MT) Attachments

#### 4.7.1. Cold Treatment Assay

To assess whether kinetochore-microtubule attachments in pyranoxanthone **2**-treated cells were cold-stable, cells in culture medium were subjected at 4 °C for 5 min and immediately processed for immunofluorescence using the follows antibodies: human anti-CREST (1:4000, gift from E. Bronze-da-Rocha, University of Porto, Portugal); mouse anti-α-tubulin (1:2500, Sigma-Aldrich Co. Ltd., Gillingham, UK). The numbers of attached or free kinetochores were counted from 5 random cells.

#### 4.7.2. MG-132 Proteasome Inhibitor Assay

To assess the ability of chromosomes to congress at equatorial zone, cells were treated with 10 μM of the proteasome inhibitor MG-132 (Sigma-Aldrich Co. Ltd., Gillingham, UK) for 1 h. Then, immunofluorescence was performed using an antibody mouse anti-α-tubulin (1:2500, Sigma-Aldrich Co. Ltd., Gillingham, UK). The number of metaphases with full alignment and metaphases with misaligned chromosomes was scored in 10 random microscope fields.

### 4.8. Terminal Deoxynucleotidyl Transferase-Mediated Nick end Labeling (TUNEL) Assay

A total of 0.15 × 10⁶ of NCI-H460 cells were treated with 1.7 μM of compound pyranoxanthone **2** for 24 h. After treatment, cells were immediately fixed in 4% paraformaldehyde (*w/v*) in PBS for 10 min, followed by PBS wash and permeabilization with 0.2% (*v/v*) Triton X-100 in PBS for 5 min. The TUNEL labeling was performed using DeadEnd Fluorometric TUNEL System (Promega, Madison, WI, USA), according to the manufacturer’s instructions. DNA was stained with 2 µg/mL DAPI in Vectashield mounting medium. The level of apoptosis was established by counting TUNEL-positive cells in a total of approximately 400 cells in 5 random fields under fluorescence microscope. The apoptotic index was calculated as the percentage of positively TUNEL-stained cells over the cells.

### 4.9. Colony Formation Assay

A total of 500 (NCI-H460) and 2000 (HPAEpiC) cells were seeded in six-well plates, allowed to attach for 24 h, and treated with 0.85 μM or 1.70 μM of pyranoxanthone **2**, 2 nM of paclitaxel or with a combination of pyranoxanthone **2** and paclitaxel. Untreated and DMSO-treated cells were also included. Forty-eight hours later, cells were washed twice with PBS and incubated in a drug-free RPMI medium for 8 days. The grown colonies were fixed for 5 min with 3.7% paraformaldehyde (*w/v*) in PBS and stained for 20 min with violet crystal (Merck) 0.05% (*w/v*) in distilled water. The number of colonies for each condition was counted in duplicate dishes from three independent experiments. The plating efficiency (PE) was calculated as the percentage of the number of grown colonies over the number of cells seeded in control. For each condition, the survival fraction was determined as the number of colonies over the number of cells seeded × 1/PE.

### 4.10. Live-Cell Imaging

For live-cell imaging experiments, 0.12 × 10^6^ NCI-H460 cells were seeded onto LabTek II chambered cover glass (Nunc, Penfield, NY, USA) containing RPMI, allowed to attach for 24 h at 37 °C with 5% CO_2_. Then, cells were treated with 1.7 μM of pyranoxanthone **2** and images were captured at 10 min intervals up to 48 h under differential interference contrast (DIC) optics, with a 63× objective on an Axio Observer Z.1 SD inverted microscope, equipped with an incubation chamber with the temperature set to 37 °C and an atmosphere of 5% CO_2_. Movies were generated from the time-lapse images using ImageJ software (version 1.44, Rasband, W.S., ImageJ, U. S. National Institutes of Health, Bethesda, MD, USA). The number of cells arrested at mitosis or in cell death was scored, based on cellular morphology. Dead cells were classified into death in mitosis (DiM) or post-mitotic death (PMD) when death occurred during or following cell division, respectively.

### 4.11. Image Acquisition and Processing

For phase contrast microscopy, a Zeiss Primo Vert microscope (Carl Zeiss, Oberkochen, Germany) and a Nikon TE 2000-U microscope (Nikon, Amsterdam, The Netherlands) with a 10× objective were used. The Nikon microscope used a DXM1200F digital camera, with Nikon ACT-1 software (Melville, NY, USA). For experiments where image acquisition was performed using fluorescence, an Axio Observer Z.1 SD microscope (Carl Zeiss, Germany) was used, coupled to an AxioCam MR3, and with the Plan Apochromatic 63×/NA 1.4 objective. The deconvolution was performed with the software AxioVision Release 4.8.2 SPC and the images were processed using ImageJ version 1.44.

### 4.12. Statistical Analysis

Statistical analysis was performed using an unpaired Student’s *t* test or two-way ANOVA with Tukey’s multiple comparisons test in the GraphPad Prism version 6 (GraphPad software Inc., CA, USA). Data are presented as the mean ± standard deviation (SD) of three independent experiments and the level of statistical significance was established considering the probabilities of * *p* < 0.05, ** *p* < 0.01, *** *p* < 0.001 and **** *p* < 0.0001.

## Supplementary Materials

The following are available online, Figure S1: ^1^H NMR spectrum of compound **8**. Figure S2: ^13^C NMR spectrum of compound **8**. Figure S3: ^1^H NMR spectrum of compound **2**. Figure S4: ^13^C NMR spectrum of compound **2**. Video S1: Untreated cell from time-lapse imaging (DIC microscopy), available online at https://youtu.be/Lfu6tMx8fks, demonstrating a normal mitosis (00h:20min). Video S2: PX2-treated cell from time-lapse imaging (DIC microscopy), available online at https://youtu.be/P9DYSUjxiIM, showing a mitotic arrest followed by death in mitosis after 10min:14h.
