# Supplementary material for: A Pyranoxanthone as a Potent Antimitotic and Sensitizer of Cancer Cells to Low Doses of Paclitaxel
Source: Molecules. 2020 Dec 10;25(24):5845. doi: 10.3390/molecules25245845 (PMC7764177; doi:10.3390/molecules25245845)
Supplement: Supplementary file 1 [file molecules-25-05845-s001.pdf]

molecules

SUPPLEMENTARY MATERIALS

Article

A Pyranoxanthone as a Potent Antimitotic and Sensitizer of Cancer Cells to Low Doses of Paclitaxel

Fábio França [1,2,†], Patrícia M. A. Silva [1,†], José X. Soares [3,†], Ana C. Henriques [1,4], Daniela R. P. Loureiro [4,5], Carlos M. G. Azevedo [5], Carlos M. M. Afonso [4,5,*] and Hassan Bousbaa [1,4,*]

1 CESPU, Institute of Research and Advanced Training in Health Sciences and Technologies (IINFACTS), University Institute of Health Sciences (IUCS), Rua Central de Gandra, 1317, 4585-322 Gandra PRD, Portugal; fabiofuff@gmail.com (F.F.); patricia.silva@cespu.pt (P.M.A.S.); A24955@alunos.cespu.pt (A.C.H.)
2 Department of Biomedical Sciences and Medicine, University of Algarve, 8005-139 Faro, Portugal
3 LAQV-REQUIMTE, Department of Chemical Sciences, Laboratory of Applied Chemistry, Faculty of Pharmacy, University of Porto. Rua de Jorge Viterbo Ferreira, 228, 4050-313 Porto, Portugal; jfxsoares@ff.up.pt
4 Interdisciplinary Center of Marine and Environmental Investigation (CIIMAR/CIMAR), Edifício do Terminal de Cruzeiros do Porto de Leixões, Av. General Norton de Matos s/n, 4050-208 Matosinhos, Porto, Portugal; dloureiro@ff.up.pt (D.R.P.L.)
5 Department of Chemical Sciences, Laboratory of Organic and Pharmaceutical Chemistry, Faculty of Pharmacy, University of Porto; 4050-313 Porto, Portugal; cgoncalves.azevedo@gmail.com
* Correspondence: hassan.bousbaa@iucs.cespu.pt (H.B.); cafonso@ff.up.pt (C.M.M.A); Tel.: +351-224-157-186 (H.B.); +351-220-428-692 (C.M.M.A)
† These authors contributed equally to this work.

Table of content

Figure S1: ^{1}H NMR spectrum of compound 8. 2
Figure S2: ^{13}C NMR spectrum of compound 8. 2
Figure S3: ^{1}H NMR spectrum of compound 2. 3
Figure S4: ^{13}C NMR spectrum of compound 2. 3
Figure S5: Lung non-tumor HPAEpiC cells are less sensitive to the antimitotic activity of pyranoxanthone **2** 4
Figure S6: Treatment with pyranoxanthone 2 (PX2) induces chromosome misalignment phenotype and leads to apoptotic cell death in MCF-7 (a and c) and A375-C5 (b and d) cancer cell lines. 5

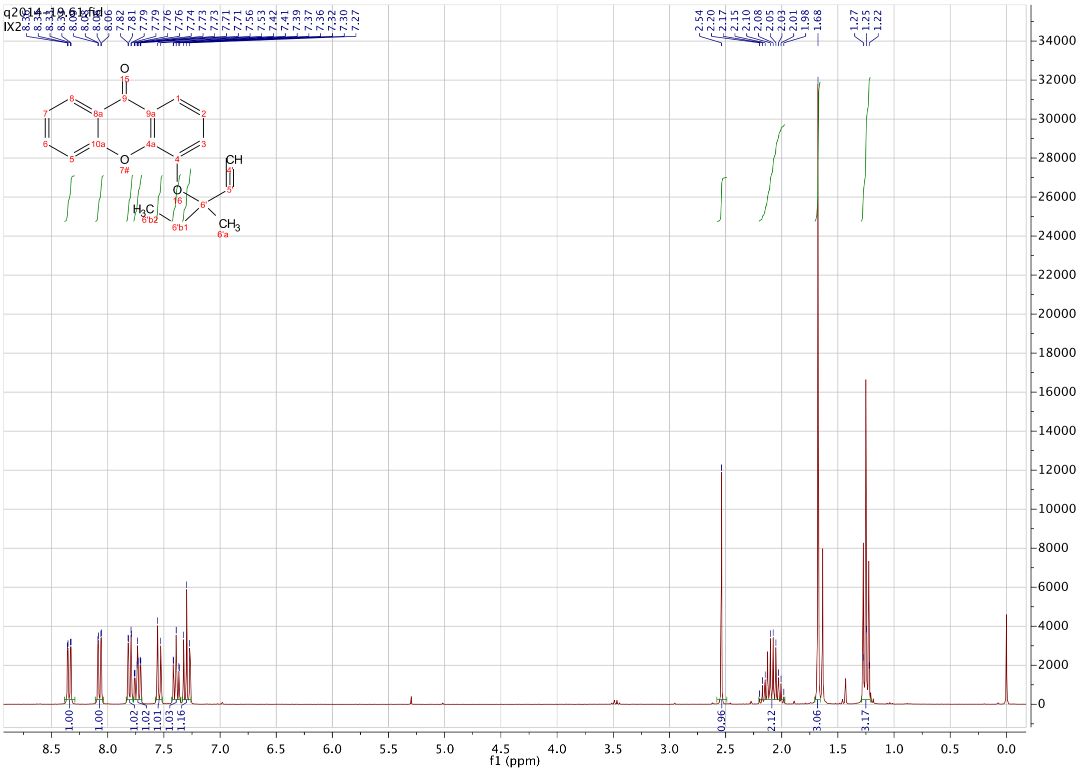

Figure 1. ^{1}H NMR spectrum of compound 8.

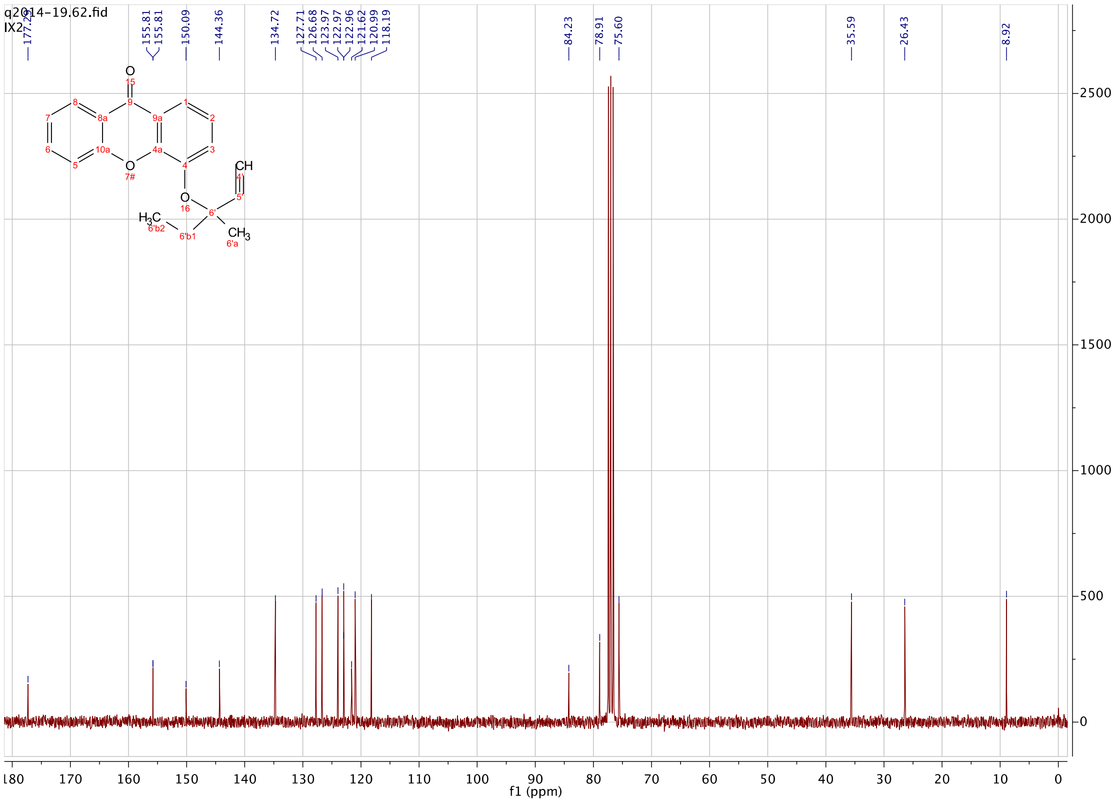

Figure 2. ^{13}C NMR spectrum of compound 8.

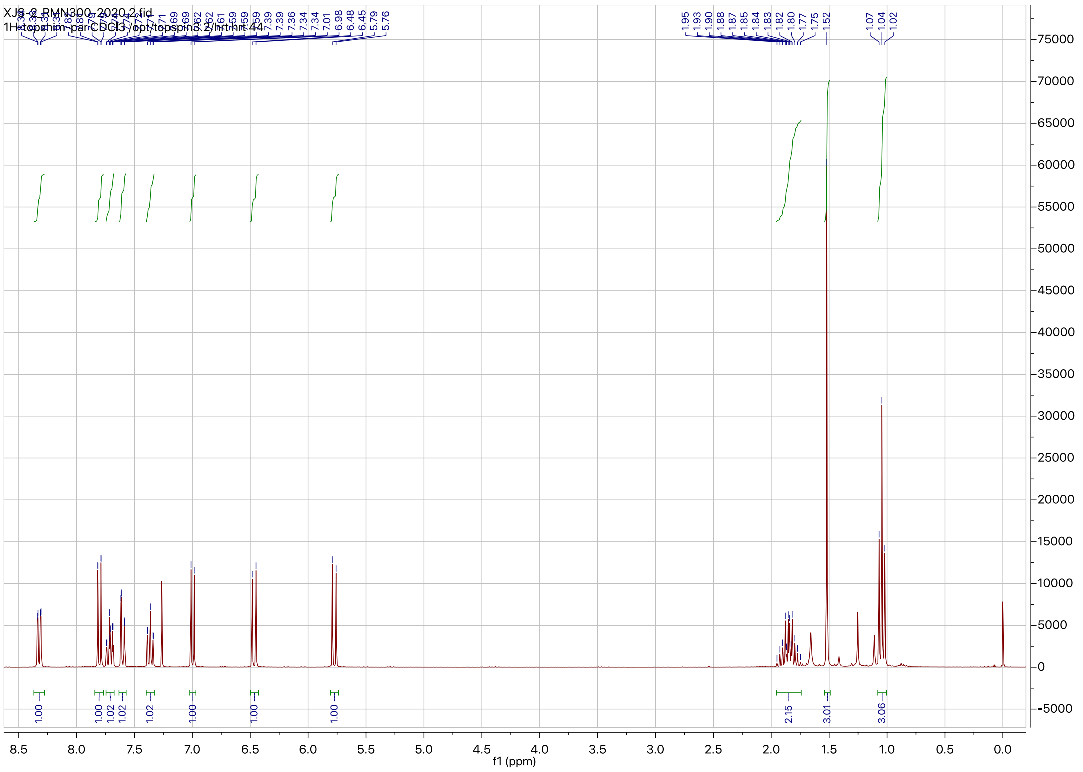

Figure 3. ^{1}H NMR spectrum of compound 2.

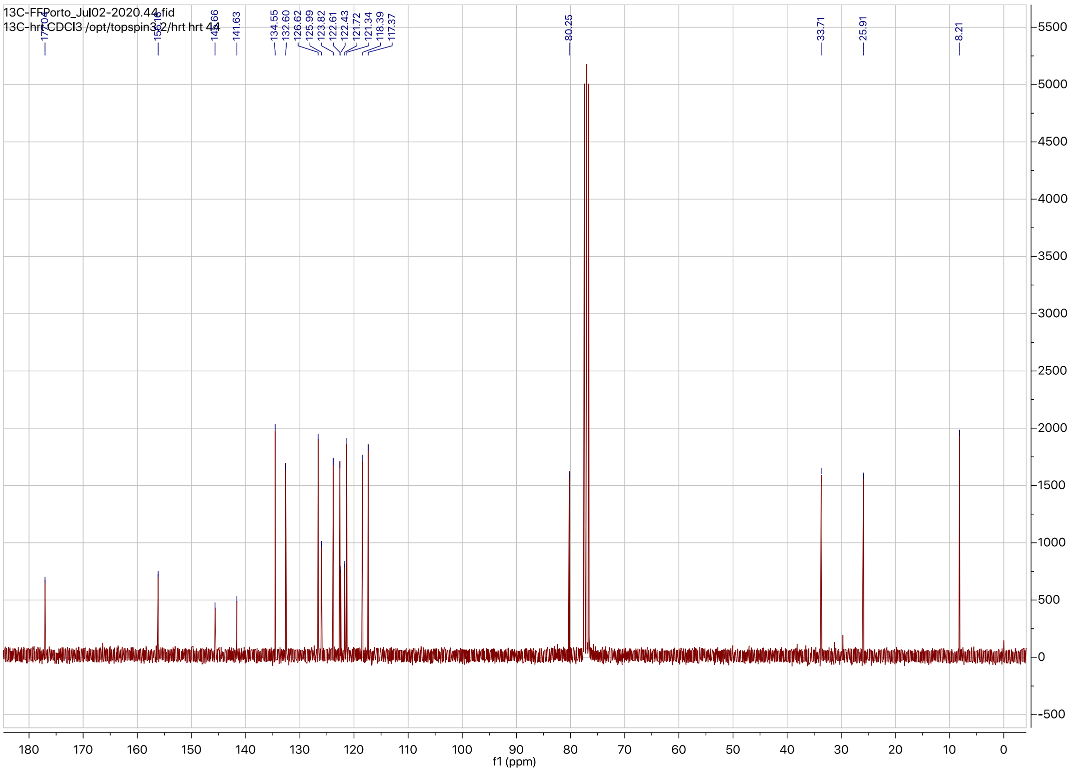

Figure 4. ^{13}C NMR spectrum of compound 2.

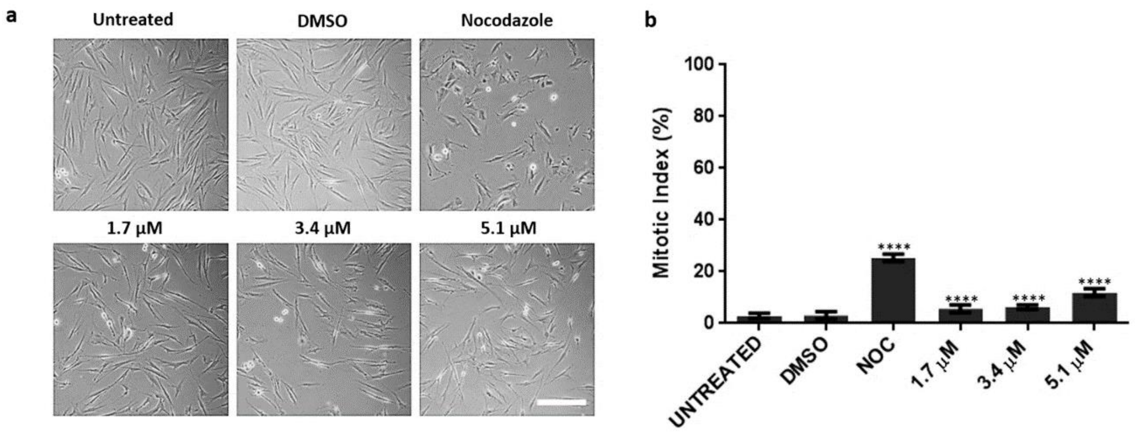

Figure S5: Lung non-tumor HPAEpiC cells are less sensitive to the antimitotic activity of pyranoxanthone **2** (PX2). (**a**) Representative phase contrast microscopy images, after 16 h treatment with pyranoxanthone 2 (PX2), at indicated concentrations. Nocodazole was used as a positive control. DMSO was used as a compound solvent control. Bar, 10 µm. (**b**) Mitotic index graph with statistical relevance of * $p < 0.05$, ** $p < 0.01$ and *** $p < 0.001$ by unpaired t-test.

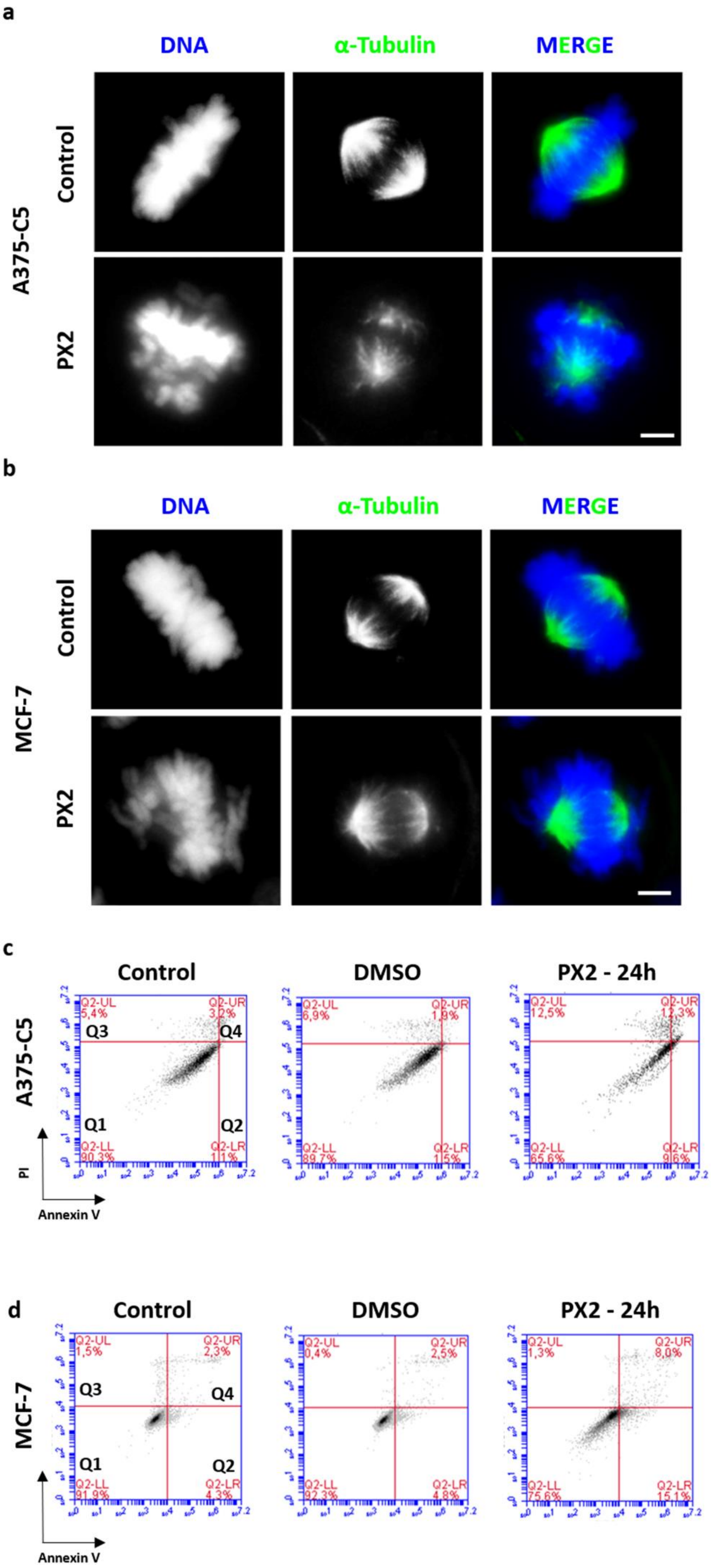
a
DNA
α-Tubulin
MERGE
A375-C5
Control
PX2
b
DNA
α-Tubulin
MERGE
MCF-7
Control
PX2
c
Control
DMSO
PX2 - 24h
A375-C5
Q2-UL 5,4%
Q2-UR 3,2%
Q3
Q4
Q1
Q2
Q2-LL 90,3%
Q2-LR 1,1%
Q2-UL 6,9%
Q2-UR 1,9%
Q2-LL 89,7%
Q2-LR 1,5%
Q2-UL 12,5%
Q2-UR 12,3%
Q2-LL 65,6%
Q2-LR 9,6%
PI
Annexin V
d
Control
DMSO
PX2 - 24h
MCF-7
Q2-UL 1,5%
Q2-UR 2,3%
Q3
Q4
Q1
Q2
Q2-LL 91,9%
Q2-LR 4,3%
Q2-UL 0,4%
Q2-UR 2,5%
Q2-LL 92,3%
Q2-LR 4,8%
Q2-UL 1,3%
Q2-UR 8,0%
Q2-LL 75,6%
Q2-LR 15,1%
Annexin V

Figure 6. Treatment with pyranoxanthone **2** (PX2) induces chromosome misalignment phenotype and leads to apoptotic cell death in MCF-7 (**a** and **c**) and A375-C5 (**b** and **d**) cancer cell lines. (**a** and **b**) Immunofluorescence images of untreated cell (Top), in metaphase, with all chromosomes aligned at equatorial zone, and PX2-treated cell (Bottom), arrested in prometaphase-like state, showing several misaligned chromosomes. Microtubules (green) were stained with anti-α-tubulin antibody and DNA (blue) with DAPI. Bar, 5 μm. (c and d) Flow cytometry analysis of apoptosis by Annexin V/PI co-staining, 24 hours after PX2 treatment. The quadrants Q were defined as Q1 = live (Annexin V- and PI-negative), Q2 = early stage of apoptosis (Annexin V-positive/PI-negative), Q3 = late stage of apoptosis (Annexin V- and PI-positive) and Q4 = necrosis (Annexin V-negative/PI-positive).

© 2020 by the authors. Submitted for possible open access publication under the terms and conditions of the Creative Commons Attribution (CC BY) license (http://creativecommons.org/licenses/by/4.0/).
